# A prospective longitudinal cohort study: evolution of GERD symptoms during the course of pregnancy

**DOI:** 10.1186/1471-230X-12-131

**Published:** 2012-09-24

**Authors:** Sara Fill Malfertheiner, Maximilian V Malfertheiner, Siegfried Kropf, Serban-Dan Costa, Peter Malfertheiner

**Affiliations:** 1 Department of Obstetrics and Gynecology, Medical Faculty of Otto von Guericke University, Gerhart-Hauptmann-Str. 35, 39108, Magdeburg, Germany; 2 Center for Pneumology, Donaustauf Hospital, Donaustauf, Germany; 3 Institute of Biometrics and Medical Informatics, Otto-von-Guericke University, Magdeburg, Germany; 4 Department of Gastroenterology, Hepatology and Infectious Diseases, Magdeburg, Germany

**Keywords:** Gastro-esophageal reflux disease, Pregnancy, Heartburn, Regurgitation, GERD symptoms

## Abstract

**Background:**

Symptoms of gastro-esophageal reflux disease (GERD) in pregnancy are reported with a prevalence of 30–80%. The aim of this study was to assess the prevalence and severity of GERD symptoms during the course of pregnancy. Furthermore current practice in medical care for GERD during pregnancy was assessed.

**Methods:**

We performed a prospective longitudinal cohort study on 510 pregnant women (mean age 28.12, SD 5.3). Investigations for reflux symptoms where based on the use of validated reflux-disease questionnaire (RDQ). Additional information was collected about the therapy. A group of non-pregnant women (mean age 24.56, SD 5.7) was included as controls. Frequency and severity of reflux symptoms were recorded in each trimester of pregnancy.

**Results:**

The prevalence of GERD symptoms in pregnant women increased from the first trimester with 26.1 to 36.1% in the second trimester and to 51.2% in the third trimester of pregnancy. The prevalence of GERD symptoms in the control group was 9.3%.

Pregnant women received medication for their GERD symptoms in 12.8% during the first, 9.1% during the second and 15.7% during the third trimester. Medications used >90% antacids, 0% PPI.

**Conclusion:**

GERD symptoms occur more often in pregnant women than in non-pregnant and the frequency rises in the course of pregnancy. Medical therapy is used in a minority of cases and often with no adequate symptom relief.

## Background

Gastro-esophageal reflux disease (GERD) is the most frequent acid-related disease as of today and occurs at all ages from childhood to advanced age [1-3]. Pregnancy has long been recognized as a condition that predisposes to GERD and GERD symptoms are known to be common in pregnant women [4,5]. Studies that have addressed the issue of GERD symptoms in pregnancy reported a prevalence between 30 and 80% [6-12]. We have previously reported a prevalence of GERD-related symptoms of 56.3%, in the third trimester of pregnancy [9].

The presentation of typical GERD symptoms during pregnancy is comparable with the adult population in general [13]. Heartburn and regurgitation are the most frequent symptoms [6,14]. The majority of pregnant GERD sufferers report exacerbation of symptoms after eating and at bedtime [15,16]. We have previously shown the negative impact of GERD symptoms on the quality of life in pregnant women and the insufficient therapy for symptom relief in late pregnancy [9].

Aim of this study was to assess the nature and prevalence of reflux symptoms in the course of pregnancy. This is the first study on GERD symptoms using validated questionnaires and using the new definition of GERD based on the Montreal criteria from 2006 which defined GERD as a condition which develops when the reflux of stomach contents causes troublesome symptoms and/or complications and allows to make the diagnosis on the basis of reflux symptoms [17].

## Methods

We conducted a prospective longitudinal cohort study to investigate the prevalence of GERD symptoms during the time course of pregnancy by using validated reflux-disease questionnaires and further gather information about the treatment patients receive.

### Subjects

Five hundred ten consecutive pregnant women in the first trimester of pregnancy and three hundred thirty non-pregnant women were recruited to participate in the study. All women were given two questionnaires: the validated German version of the Reflux Disease Questionnaire (RDQ) [18] and a self-administered questionnaire. The pregnant women were followed up and repeated to fill out the RDQ in the second and third trimester of pregnancy. Pregnant women were recruited from the University clinic and private clinics in and around Magdeburg, Germany (population approximately 300 000 people), the group of non pregnant women was recruited at the University campus and in the city of Magdeburg. The study was approved by the ethics committee of the University of Magdeburg and conducted based on the guidelines of the Helsinki declaration.

### Definition

The diagnosis of symptomatic GERD was made according to the Montreal criteria 2006, where GERD is defined as a condition which develops when the reflux of stomach contents causes troublesome symptoms and/or complications [17]. Mild symptoms occurring 2 or more days a week, or moderate/severe symptoms occurring more than 1 day a week are often considered troublesome by patients [17].

Extra-esophageal symptoms were not considered for the diagnosis of GERD in our population because of lack of specificity.

### Questionnaires

The RDQ comprises 12 questions; six are related to symptom frequency and six to symptom severity occurring during the previous week [18]. Symptoms evaluated in the RDQ were: burning behind the breastbone, pain behind the breastbone, upper stomach burning, upper stomach pain, acid taste in the mouth and regurgitation. Symptom frequency and severity were measured on six-point scales; from no occurrence to daily, and from none to very severe, respectively. GERD was diagnosed according to the definition mentioned above.

In the self-administered questionnaire, age, weight before pregnancy, height, parity, medication intake (including prescribed or over-the-counter use of antacids), additional diseases, working status, level of education and consumption of caffeine, tobacco and alcohol were recorded for each patient. BMI was based on the height and weight before pregnancy. Additionally, questions were given about the patient’s GERD symptoms before pregnancy as well as the patient’s mother’s history in relation to GERD during her pregnancy and chronic heartburn. Exclusion criteria included operations or malformations of the gastrointestinal tract, chronic inflammatory bowel diseases and high-risk pregnancies.

### Statistical analysis

Results are expressed as mean, unless otherwise indicated. All data was processed with the statistical analysis program SAS (Cary, NC, USA). P-values were calculated using Unpaired *t*-test or Fishers exact test as indicated.

## Results

### Demographic characteristics

**Figure 1:**
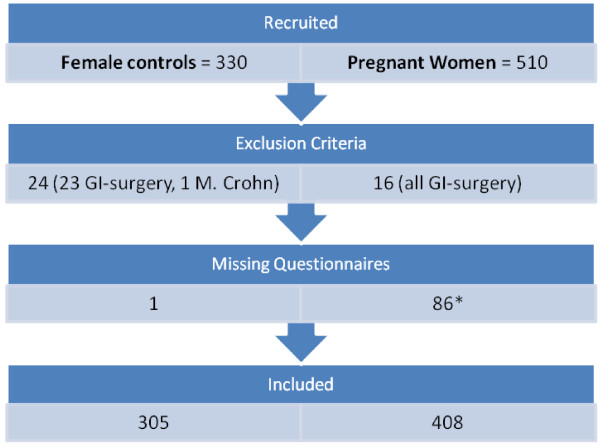
**Flowchart of participants across the study.** * 54 missing RDQ 1. Trimester, 9 missing RDQ 2. +3. Trimester, 4 missing RDQ 2. Trimester, 19 missing RDQ 3. Trimester.

A total of 840 (510 pregnant) women were recruited and returned written informed consent, Figure 1 shows the flow of participants across the study. Seven hundred thirteen women were finally included, 408 pregnant and 305 non-pregnant. The age of pregnant women ranged from 14 to 43 years (mean 28.12 ± 5.3), in the non pregnant women from 17 to 47 years (mean 24.56 ± 5.7). BMI ranged from 15.8 to 41.3 (mean 23,74 ± 4,5) in pregnant women and from 16.8 to 36.4 (mean 22.12 ±3.2) in non pregnant women. 61.3% of the pregnant were primipara, in the group of non-pregnant women 16.1% have been pregnant before. Patient demographics are shown in Table 1.

**Table 1:** Demographic data

**Variables**	**Pregnant**	**Non-pregnant**	**p-value**
Number of patients	408	305	
Mean age (years) (SD)	28.12 (5.3)	24.56 (5.7)	< 0.0001
Mean weight (SD)	66.3 (13.9)	62.2 (9.8)	<0.0001
Mean height (SD)	167.0 (6,3)	167.7 (6.6)	0.189
Mean BMI (SD)	23,74 (4,5)	22,12 (3,2)	<0.0001
Smokers	19.8%	22.2%	0.499
Coffee consumer	68.3%	65.4%	0.458

### GERD symptoms

The most frequent symptoms in pregnant and non-pregnant women were acid taste in mouth and regurgitation. In the third trimester of pregnancy 40.7% suffered from regurgitation at least once a week. Regurgitation occurred in 3.6% of non-pregnant women more than once a week. Of the pregnant women 12.5% had heartburn at least once a week during their first trimester, 21.5% during the second and 35.3% during the third trimester of pregnancy. Heartburn al least once a week was experienced by 1.6% of non pregnant women. Daily heartburn occurred in 10.1% of women in the third trimester.

Every symptom recorded occurred significantly more often in pregnant than in non-pregnant women (Table 2). And the frequency of all individual symptoms increased significantly from one trimester to the next and presented the highest frequency in the third trimester (Table 2).

**Table 2:** Frequency of symptoms (all values given in %)

**Symptom**	**Burning behind breastbone**				**Pain behind breastbone**				**Upper stomach burning**				**Upper stomach pain**				**Acid taste in mouth**				**Regurgitation**			
None	82,8	71,4	55,8	96,1	89,5	83,6	77,4	92,4	85,1	79,2	68,7	94,8	89,7	82,8	75,5	83,4	75,9	69,1	53,1	84,6	73,4	66,0	48,2	82,6
<1x/week	4,5	7,1	8,9	2,3	6,0	7,2	8,2	5,3	5,7	5,9	7,7	3,9	5,3	7,3	7,5	9,9	7,2	8,2	9,8	10,2	9,5	9,1	11,1	13,8
>1x/week	2,7	6,9	8,6	0,7	1,7	3,0	5,7	1,3	2,7	4,7	6,5	1,0	2,8	3,0	7,3	4,3	5,5	7,2	8,8	3,3	5,2	8,1	10,3	2,3
2–3x/week	7,2	7,1	10,6	1,0	2,0	3,5	3,5	0,0	4,0	6,4	7,9	0,3	1,8	4,5	6,3	1,3	7,2	8,4	13,0	1,3	5,2	10,8	12,0	1,3
4–5x/week	0,5	3,0	5,9	0,0	0,2	0,5	1,7	0,7	1,7	1,5	3,2	0,0	0,5	1,0	1,0	0,7	1,7	2,0	7,6	0,3	3,5	3,0	8,4	0,0
Daily	2,2	4,4	10,1	0,0	0,5	2,2	3,5	0,3	0,7	2,2	6,0	0,0	0,0	1,5	2,5	0,3	2,5	5,2	7,6	0,0	3,2	3,0	10,1	0,0
	1T.	2T.	3T.	C.	1T.	2T.	3T.	C.	1T.	2T.	3T.	C.	1T.	2T.	3T.	C.	1T.	2T.	3T.	C.	1T.	2T.	3T.	C.

Regarding the severity of the symptoms beside acid taste in mouth and regurgitation also a burning sensation behind the breastbone were symptoms perceived as most bothersome (Table 3). Acid taste in mouth was reported as severe or very severe in 4.3%, 8.4% and 18.3% from the first to the third trimester respectively. The severity of symptoms was significantly higher in pregnant than in non-pregnant women and increased steadily during the course of pregnancy (Table 3). At least one symptom was experienced severe or very severe by 29.2% of women in the third trimester compared to 13.2% in the second and 11.3% in the first trimester. The prevalence and severity of the symptoms is shown in detail in Tables 2 and 3.

**Table 3:** Severity of symptoms (all values given in %)

**Symptom**	**Burning behind breastbone**				**Pain behind breastbone**				**Upper stomach burning**				**Upper stomach pain**				**Acid taste in mouth**				**Regurgitation**			
None	86,6	75,5	57,7	96,8	91,8	85,5	80,1	94,0	88,0	81,7	71,4	96,5	90,4	84,0	78,7	86,5	80,2	73,6	55,4	85,5	77,7	70,3	51,6	85,2
Very mild	2,2	4,3	7,0	1,1	3,0	5,5	4,9	2,5	3,3	4,4	3,8	2,1	3,8	5,2	6,8	3,5	4,1	5,4	7,5	7,1	3,8	6,0	7,3	7,8
Mild	1,6	4,9	6,0	0,7	1,6	2,5	4,6	1,8	2,2	3,0	5,4	0,4	2,5	1,7	2,7	4,6	4,6	4,3	8,1	4,3	4,9	6,8	8,1	4,6
Moderate	3,8	6,0	11,7	1,1	2,2	3,6	5,2	1,4	2,2	5,7	7,1	1,1	2,2	5,5	4,9	4,6	6,8	8,2	10,8	3,2	6,0	7,1	15,9	1,4
Severe	3,5	4,9	8,4	0,4	0,8	1,4	1,6	0,4	3,0	3,0	6,0	0,0	0,8	2,2	4,4	0,7	2,4	4,6	11,8	0,0	3,5	4,9	7,8	1,1
Very severe	2,2	4,3	9,2	0,0	0,5	1,6	3,5	0,0	1,4	2,2	6,3	0,0	0,3	1,4	2,5	0,0	1,9	3,8	6,5	0,0	4,1	4,9	9,4	0,0
	1T.	2T.	3T.	C.	1T.	2T.	3T.	C.	1T.	2T.	3T.	C.	1T.	2T.	3T.	C.	1T.	2T.	3T.	C.	1T.	2T.	3T.	C.

### GERD diagnosis

The diagnosis of GERD was based on typical reflux symptoms, either mild symptoms occurring two or more days a week, or moderate/severe symptoms occurring more than one day a week. Symptoms referring to upper stomach pain and upper stomach burning recorded with the RDQ as well were excluded for GERD diagnosis as they are considered to be nonspecific for GERD [18].

**Figure 2:**
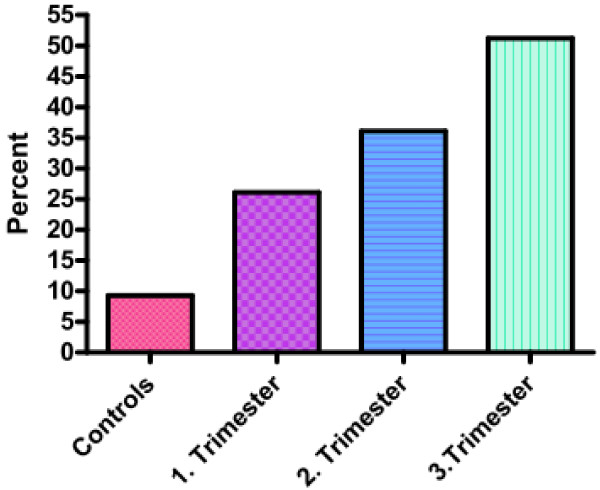
Prevalence of GERD in the trimesters of pregnancy and a non-pregnant control group (values given in percent %).

The symptom based diagnosis of GERD found a prevalence of 26.1% in the first trimester, 36.1% in the second trimester and 51.2% in the third trimester of pregnancy (Figure 2). In non pregnant women the prevalence of GERD was 9.3%.

### GERD therapy

The use of medication for neutralizing or suppressing gastric acid was recorded with a self administered questionnaire. In the first trimester of pregnancy 12.8% of women reported to use acid neutralizing medications, in the second trimester 9.1% and in the third 15.7%. In the third trimester only one patient received an H2receptorantagonist, all others utilized antacids, none received PPI. The distribution of used medications during pregnancy is shown in Table 4. In the group of non-pregnant women 3.3% reported intake of antacids and PPI. GERD symptoms in pregnant women on antacids increased during pregnancy in the same magnitude as in those with no medication. The evolution of heartburn and regurgitation is shown in Table 5.

**Table 4:** Medications taken for reflux symptoms during pregnancy

**Medications**	**1. trimester**	**2. trimester**	**3. trimester**
Calcium carbonate, Magnesium carbonate (Rennie®)	44	46.5	49.4
Magaldrate (Riopan®, Magaldrat®)	39	34.9	26.5
Algedrate, magnesium hydroxide (Maaloxan®)	8.5	14	8.4
Hydrotalcide (Talcid®)	3.4	-	2.4
Almasilate (Simagel®)	3.4	-	6
Sodium bicarbonate (Bullrich Salz®)	1.7	2.3	1.2
Sodium bicarbonate, Calcium carbonate, alginic acid (Gaviscon®)	-	2.3	4.8
Ranitidine (Ranitic®)	-	-	1.2

All values are given in percent, brand names are given in brackets.

**Table 5:** Frequency and severity of regurgitation in patients receiving Antacids in all trimesters of pregnancy

**Frequency**	**1T.**	**2T.**	**3T.**	**Severity**	**1T.**	**2T.**	**3T.**
None	27,3	13,0	17,4	None	28,57	12,50	0,0
<1x/week	27,3	17,4	8,7	Very mild	14,29	25,00	12,50
>1x/week	18,2	43,5	34,8	Mild	28,57	12,50	25,00
2–3x/week	13,6	13,0	17,4	Moderate	14,29	25,00	12,50
4–5x/week	9,1	8,7	4,3	Severe	0,0	12,50	12,50
Daily	4,5	4,3	17,4	Very severe	14,29	12,50	37,50

n = 23 (all values given in %).

While using antacids 14.29% of women reported regurgitation as very severe, these women continued the intake of antacids throughout their pregnancy and 37.5% of them reported regurgitation as very severe in the third trimester.

## Discussion

GERD symptoms are very frequent in pregnant women and they increase in severity during the course of pregnancy. The novelty of our study is the methodology employed for the assessment of symptoms. The study is the largest longitudinal study using validated questionnaires to asses GERD symptoms in pregnant women. The overall prevalence of 26,1%; 36,1% and 51,2% in the trimesters of pregnancy display the magnitude of the problem and is in accordance and extends the results of previous studies [6,10-12]. The pathophysiologic mechanisms involved in the abnormal gastric reflux during pregnancy are decreased lower esophageal sphincter pressure and alteration in gastrointestinal transit due to hormone changes, and increased intra-abdominal pressure secondary to the enlarged gravid uterus [4,19].

GERD impairs the quality of life, and the negative impact is felt as a severe threat when symptoms are experienced severe or very severe. An aspect of importance is that more than every third woman suffers from severe GERD symptoms during pregnancy, but not even every fifth woman with symptoms receives acid neutralizing/suppressive medication. There is good reason for the cautions use of medical therapy during pregnancy because of the fear of having side effects for the unborn life at the back of one’s mind. Though we do not exactly know how influencing the untreated suffering of the mothers is. The standard medication for GERD in the non-pregnant population is proton pump inhibitors (PPIs) which were shown to be effective and well tolerated. In contrast to the non-pregnant population a step-up algorithm is recommended during pregnancy [5].

There is prospective data on PPIs taken during the first trimester of pregnancy, that showed no increased risk of major birth defects [20]. PPIs can be considered as safe drugs during pregnancy [21,22]. In most pregnant women reflux symptoms can be managed by lifestyle modifications and intermittent use of antacids. However in women with severe symptoms PPIs should be the treatment of choice as they are most effective with no safety concerns. Omeprazole is the best studied PPI in pregnancy [22].

## Conclusion

Every second women suffers from GERD during pregnancy. Heartburn, regurgitation, and acid taste in mouth are bothersome symptoms affecting the women’s quality of life. Not even half of the women with severe or very severe symptoms are treated adequately. Our data and the last publications on safety of PPIs during pregnancy should be implemented in novel recommendations for GERD therapy during pregnancy.

## Competing interests

The authors declare that they have no competing interests.

## Authors’ contribution

SFM has made substantial contributions to conception and design of the study, and interpretation of the data, further she was responsible for the data collection. MM participated in the analysis and interpretation of the data and helped to draft the manuscript. SK was responsible for the statistical analysis of the data. SC has critically revised the manuscript and has added important intellectual content. PM has been involved in drafting the manuscript and gave final approval of the version to be published. All authors read and approved the final manuscript.

## Pre-publication history

The pre-publication history for this paper can be accessed here:

http://www.biomedcentral.com/1471-230X/12/131/prepub
